# Analysis of criteria for treatment initiation in patients with progressive chronic lymphocytic leukemia

**DOI:** 10.1038/s41408-017-0044-5

**Published:** 2018-01-16

**Authors:** Pablo Mozas, Alfredo Rivas-Delgado, Tycho Baumann, Neus Villamor, Valentín Ortiz-Maldonado, Marta Aymerich, Dolors Costa, Alba Navarro, Eva Giné, Armando López-Guillermo, Emili Montserrat, Julio Delgado

**Affiliations:** 1 Department of Hematology, Institute of Hematology and Oncology, Hospital Clinic, Institut d’Investigacions Biomèdiques August Pi i Sunyer (IDIBAPS), University of Barcelona, Barcelona, Spain; 2 Department of Pathology, Hematopathology Unit, Hospital Clínic, IDIBAPS, University of Barcelona, Barcelona, Spain; 3 CIBERONC, Madrid, Spain

Chronic lymphocytic leukemia (CLL) is an indolent and incurable disorder characterized by the progressive accumulation of monoclonal B-cells in blood, bone marrow, and lymphoid organs. Since early intervention before the disease becomes symptomatic does not procure any benefit to patients^1^, treatment is only indicated in case of active disease, which is defined by the presence of marrow failure (MF), progressive lymphadenopathy, or splenomegaly (i.e., lymphoid mass (LM)), refractory immune cytopenia, general symptoms, or short lymphocyte doubling time^2–5^. These criteria are largely based on clinical experience and have never been validated empirically. Moreover, some of these criteria correlate with clinico-biological features of the disease. For instance, lymphadenopathy is associated with 11q deletion and increased risk of Richter’s transformation (RT)^6^. In addition, infiltrative cytopenia (as opposed to immune cytopenia) is associated with short survival^7^ and also with advanced age, which in turn predicts for a shorter survival^8^. The aims of this study were to evaluate the clinico-biological characteristics and outcome of patients with progressive CLL depending on the criterion that prompted first-line treatment.

After approval by the Institution’s Review Board, we selected all consecutive patients from our institutional database requiring frontline therapy from 1978 to 2014, provided that they could be classified according to treatment criteria. These were: (i) progressive MF; (ii) massive or progressive lymphadenopathy; (iii) massive or progressive splenomegaly; (iv) autoimmune anemia or thrombocytopenia unresponsive to standard therapy; (v) presence of general symptoms; and (vi) short lymphocyte doubling time^2–5^. Patients with progressive lymphadenopathy and those with progressive splenomegaly were difficult to separate from each other and were consequently grouped as “LM.” Patients treated due to anemia or thrombocytopenia had their clinical records thoroughly investigated to correctly assign them to either MF or immune cytopenia. Fluorescent in situ hybridization (FISH) aberrations were classified following Döhner’s hierarchical model and further grouped in low risk (normal, 13q− or +12) and high risk (11q− or 17p−)^9^. *IGHV* somatic mutations were evaluated following ERIC recommendations^10^, and *NOTCH1*, *SF3B1*, and *TP53* mutations using previously described methods^11^.

Associations between baseline features were assessed by Fisher’s exact test or Wilcoxon rank-sum test. Richter’s transformation was calculated from frontline therapy using cumulative incidence curves, and the effect of each covariate was assessed using Gray’s test. Overall survival (OS) was estimated from frontline therapy using Kaplan–Meier curves, and the effect of each covariate was assessed using the log-rank test. Complete-case multivariate analyses were modeled using the Cox regression equation, and the proportional hazards assumption was tested using Schönfeld residuals^12^. Apart from treatment criteria, the following covariates were evaluated: *IGHV* mutation status (mutated vs. unmutated); age (continuous); beta_2_-microglobulin (B2M, continuous), and FISH aberrations (low vs. high risk). Relative survival was computed using Spanish population data. All calculations were performed using R (version 3.2.4). Adjusted *P* values <0.05 (Benjamini–Hochberg correction) were considered significant.

We identified 567 consecutive patients who received CLL-specific therapy, but information on treatment criteria was available in 530 (93%) of them (Supplemental Table 1). Median age was 62 years (range, 22–93) at diagnosis and 65 years (range, 22–95) at frontline therapy, and 63% of patients were male. Treatment consisted of alkylating agents in 58%, purine analogs in 21%, purine analogs plus rituximab in 16%, and other therapies in the remaining 5%. Treatment eras were 1978–1990 in 14%, 1990–2000 in 40%, and >2000 in 46%. Median follow-up from first-line therapy was 94 (range, 1–433) and 64 (range, 1–433) months for survivors and the entire population, respectively. Treatment criteria were MF in 31%, LM in 72%, refractory immune cytopenia in 3%, general symptoms in 19%, and short lymphocyte doubling time in 29% (Supplemental Fig. 1). In total, 266/530 (50%) patients had a single criterion, while the remaining 264 patients had two or more criteria for treatment initiation. Of note, 506/530 (95%) patients were treated due to MF, LM, or both, with or without additional criteria, while only 24 (5%) patients received therapy due to refractory immune cytopenia, general symptoms, short lymphocyte doubling time, or a combination of them (Supplemental Fig. 1). Consequently, we focused our analysis on the 506 patients treated due to MF, LM, or both. Patients who received therapy due to both MF and LM (47/506 (9%)) were classified as MF following the same logic behind Binet and Rai staging systems^13,14^.

Compared to LM patients, MF patients were significantly older, had a significantly higher B2M concentration, and received alkylating agents more frequently (Supplemental Table 1). As expected, the MF population had a significantly lower hemoglobin concentration and platelet count at diagnosis, which was associated with a more advanced disease in terms of Rai or Binet clinical stages. There were a few cases of Binet stage C (*n* = 7) and Rai stage III–IV (*n* = 8) disease in the LM population, which were due to immune cytopenia. The LM group showed a higher proportion of adverse prognostic features compared to the MF group, including unmutated *IGHV* genes (*P* = 0.001), high ZAP70 expression (*P* = 0.001), and presence of 11q deletion (*P* = 0.05), although biomarkers were only available in around 60% of patients. In contrast, 13q deletions were more frequently detected in patients with MF (*P* = 0.03). Missing results were equally distributed across both groups (*P* = 0.15).

The median OS of the entire population was 77 months (95% confidence interval (CI): 71–83) from first-line therapy and 108 months (95% CI: 102–117) from diagnosis. When we evaluated each treatment criterion individually (i.e., patients who had that criterion vs. those who did not), we observed that both MF and LM had a significant impact on OS (*P* < 0.001 for both comparisons, Fig. 1a, d). We also observed a trend toward a shorter survival for patients treated due to immune cytopenia (Fig. 1e, *P* = 0.06). Of note, MF was associated with a shorter OS compared to the other treatment criteria pooled together, whereas LM had the opposite effect (i.e., it was associated with a longer OS compared to the other criteria pooled together). Moreover, since MF and LM were present in 95% of patients, and Fig. 1a looked like the specular reflection of Fig. 1d, we combined both criteria into one covariate called “treatment criteria” (LM vs. MF). In this subset comprising 95% of the original population, the median OS for MF patients was 63 months (95% CI: 48–72) compared to 89 months (95% CI: 80–106) for LM patients (*P* < 0.001, Fig. 2a). Treatment criteria (MF vs. LM) remained a significant predictor of OS after adjusting by age (70 years or less vs. >70 years; *P* < 0.001), B2M (2.4 mg/L or less vs. >2.4 mg/L; *P* = 0.002), *IGHV* mutational status (mutated vs. unmutated; *P* < 0.001), FISH aberrations (low vs. high risk; *P* = 0.012), or frontline therapy (alkylating agents vs. purine analogs; *P* < 0.001). By multivariate analysis, three covariates had an independent impact on OS: age (hazard ratio (HR) 1.04, 95% CI: 1.03–1.05, *P* < 0.001), B2M (HR 1.07, 95% CI: 1.03–1.12, *P* < 0.001), and treatment criteria (HR 1.29, 95% CI: 1.01–1.66, *P* = 0.041). B2M violated the proportional hazards assumption (*P* = 0.026) and was removed from the model. When the multivariate analysis was repeated including B2M as a stratum, we confirmed that both age (HR 1.04, 95% CI: 1.03–1.06, *P* < 0.001) and treatment criteria (HR 1.38, 95% CI: 1.02–1.88, *P* = 0.038) retained their statistical significance. Other covariates, such as *IGHV* mutation status or FISH aberrations were not included in the model because they were only available in around 60% of patients.

**Fig. 1:**
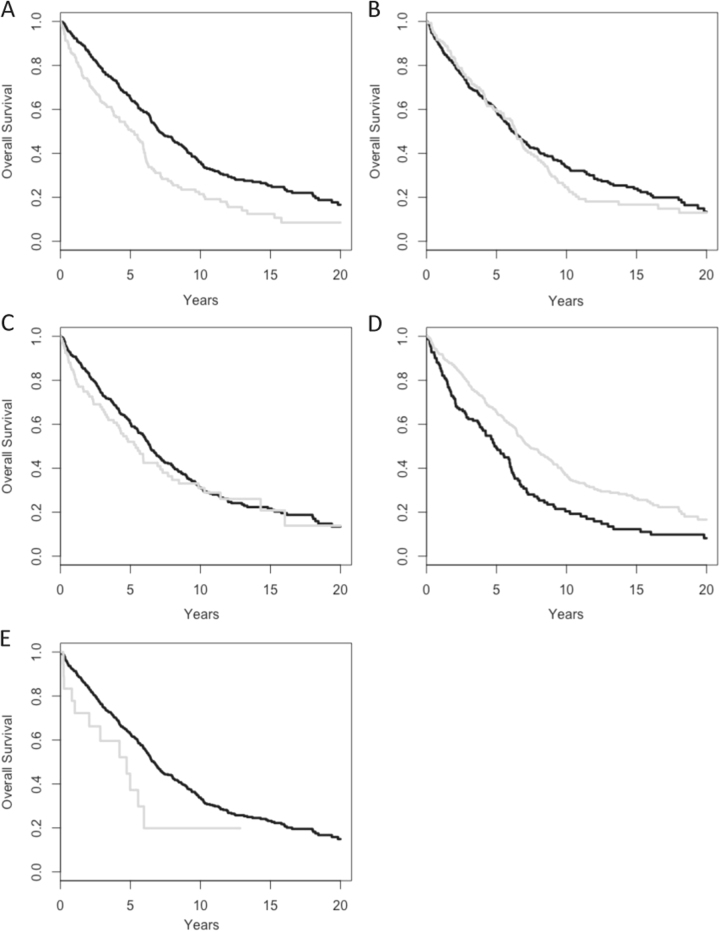
Overall survival for the entire patient population according to each treatment criterion individually. **a** Marrow failure (*P* < 0.001); **b** short doubling time (*P* = 0.4); **c** general symptoms (*P* = 0.4); **d** lymphoid mass (*P* < 0.001); and **e** autoimmune hemolytic anemia (*P* = 0.06). In all plots, the gray curve represents the presence and the black curve the absence of that particular criterion. *P* values were adjusted using the Benjamini–Hochberg method

**Fig. 2:**
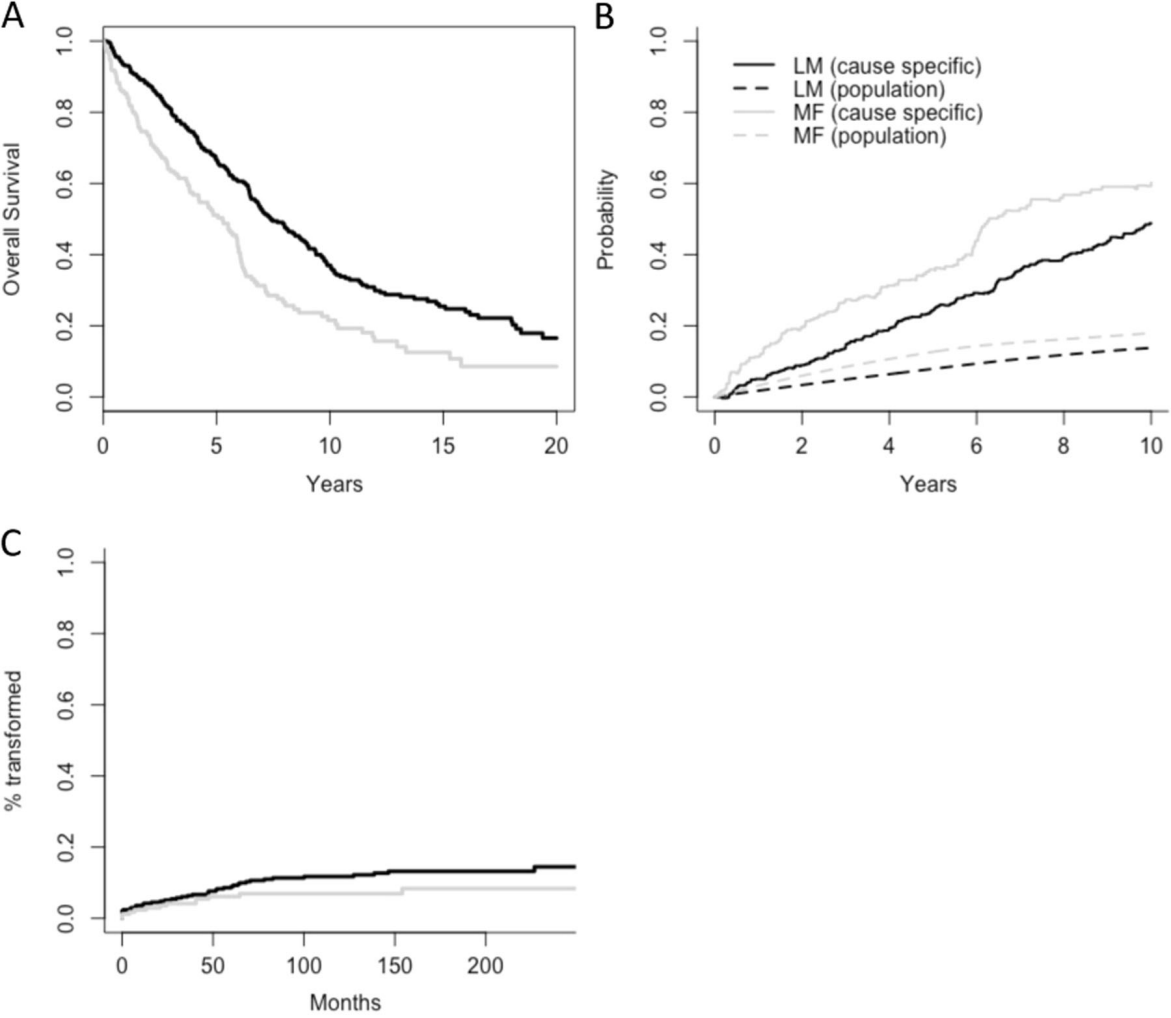
Overall survival, CLL-specific mortality and incidence of Richter's transformation according to treatment criteria. **a** Overall survival according to treatment criteria, considering only patients treated because of marrow failure (MF), lymphoid mass (LM), or both (95% of the entire population). Patients who fulfilled both criteria were assigned to the MF group (*P* < 0.001). **b** CLL-specific mortality curves according to treatment criteria. Normal population mortality curves are also plotted for reference. Notice that the MF-reference mortality is slightly higher than the LM-reference mortality because this population is significantly older. **c** Cumulative incidence of Richter’s transformation according to treatment criteria (*P* = 0.20). MF patients are depicted in gray and LM patients are depicted in black

Since the studied population spanned many decades, we evaluated whether the impact of treatment criteria was independent of patient’s age, sex, or calendar date by modeling the relative survival of our cohort. Both age and treatment criteria remained statistically significant (*P* < 0.001 and *P* = 0.0233, respectively). CLL-specific and normal population mortality plots confirmed that patients requiring therapy had a significantly higher mortality compared to the normal population, and that MF patients had a significantly higher CLL-specific mortality than LM patients (Fig. 2b).

Since bulky lymphadenopathy has been defined as a factor linked to the development of RT, we evaluated whether treatment criteria (LM vs. MF) was associated with that outcome. However, the cumulative incidence of transformation was not significantly affected by this covariate. As such, the incidence of transformation 10 years after treatment initiation was 12% (95% CI 9–16%) for the LM cohort compared to 7% (95% CI 4–12% for the MF cohort (*P* = 0.20, Fig. 2c)). There was borderline significance for high-risk FISH aberrations (11q− or 17p−, *P* = 0.09) and *IGHV* mutational status (*P* = 0.12), and a clear statistical significance for *NOTCH1* mutations (*P* < 0.001, Supplemental Fig. 2), which is in keeping with previous reports^15^.

In summary, this study shows that patients with CLL treated because of LM have a better outcome than those in whom therapy is initiated due to MF. This retrospective analysis derives from a large patient population treated over a long period of time with standard chemotherapy or, more recently, chemo(immuno)therapy regimens available at each time period. Whether the observations presented here hold for novel therapies whose main therapeutic effect is observed in lymph nodes (i.e., B-cell receptor inhibitors) should be prospectively investigated.

## Electronic supplementary material

Supplemental material

Supplemental Figure 1
