# Supplementary material for: Analysis of criteria for treatment initiation in patients with progressive chronic lymphocytic leukemia
Source: Blood Cancer J. 2018 Jan 16;8(1):10. doi: 10.1038/s41408-017-0044-5 (PMC5802533; doi:10.1038/s41408-017-0044-5)
Supplement: Supplementary file 1 — Supplemental material [file 41408_2017_44_MOESM1_ESM.docx]

**SUPPLEMENTAL FIGURES: Analysis of criteria for treatment initiation in patients with progressive chronic lymphocytic leukemia** (Mozas et al.)

**Supplemental Table 1: Patients’ baseline characteristics according to treatment indication**

| **Main indication for treatment** | **Marrow failure**  **(n = 171)** | **Lymphoid mass**  **(n = 335)** | ***P value*** |
| --- | --- | --- | --- |
| Age at diagnosis in years, median (range) | 66 (38-93) | 58 (22-89) | < 0.001 |
| Male sex, n (%) | 110 (64) | 212 (63) | 0.81 |
| Binet stage C at diagnosis, n (%) | 40 (24) | 7 (2) | < 0.001 |
| Rai stage III-IV at diagnosis, n (%) | 48 (29) | 8 (2) | < 0.001 |
| Hemoglobin concentration (g/L), median (range) | 130 (46-181) | 137 (87-185) | < 0.001 |
| Lymphocyte count (x10^9^/L), median (range) | 16.7 (5.2-375) | 16.8 (5.1-410) | 0.25 |
| Platelet count (x10^9^/L), median (range) | 156 (7-408) | 186 (57-470) | < 0.001 |
| Beta_2_-microglobulin (mg/L), median (range) | 3.2 (1.1-12.8) | 2.5 (0.4-31) | < 0.001 |
| FISH abnormalities [n=338 (67%)] |  |  | 0.18 |
| Normal, n (%) | 23 (23) | 68 (28) |  |
| del(13q), n (%) | 44 (45) | 77 (32) |  |
| +12, n (%) | 15 (15) | 38 (16) |  |
| del(11q), n (%) | 10 (10) | 42 (17) |  |
| del(17p), n (%) | 7 (7) | 17 (7) |  |
| High ZAP70 expression, n (%) [n=331 (66%)] | 42 (48) | 135 (58) | 0.001 |
| Unmutated *IGHV* genes, n (%) [n=266 (53%)] | 36 (46) | 144 (69) | 0.001 |
| Mutated *NOTCH1* gene, n (%) [n=283 (56%)] | 9 (11) | 41 (20) | 0.10 |
| Mutated *SF3B1* gene, n (%) [n=260 (50%)] | 6 (8) | 33 (17) | 0.12 |
| Mutated *TP53* gene, n (%) [n=235 (45%)] | 6 (9) | 20 (11) | 0.72 |
| Treatment |  |  | <0.001 |
| Alkylating agents, n (%) | 114 (67) | 171 (51) |  |
| Purine analogs, n (%) | 22 (13) | 92 (27) |  |
| Purine analogs plus rituximab, n (%) | 23 (13) | 62 (19) |  |
| Others, n (%) | 12 (7) | 10 (3) |  |
| Treatment era |  |  | 0.75 |
| 1978-1990 | 25 (15) | 42 (12) |  |
| 1990-2000 | 63 (37) | 132 (39) |  |
| 2000-2015 | 83 (48) | 161 (48) |  |
| Follow-up from therapy in months, median (range) | 46 (1-291) | 76 (1-433) | < 0.001 |

**Supplemental Figure 1: Venn diagram showing the entire patient population according to treatment criteria**. Abbreviations: MF, marrow failure; SLDT, short lymphocyte doubling time; GS, general symptoms; LM, lymphoid mass; IC, immune cytopenia.

**Supplemental Figure 2:** **Cumulative incidence of Richter’s transformation according to (A) *IGHV* mutational status (black = unmutated; grey = mutated); (B) *NOTCH1* mutations (black = unmutated; grey = mutated) and (C) FISH aberrations (black = low risk; grey = high risk).**
