# Supplementary figures and images for: Analysis of criteria for treatment initiation in patients with progressive chronic lymphocytic leukemia
Source: Blood Cancer J. 2018 Jan 16;8(1):10. doi: 10.1038/s41408-017-0044-5 (PMC5802533; doi:10.1038/s41408-017-0044-5)

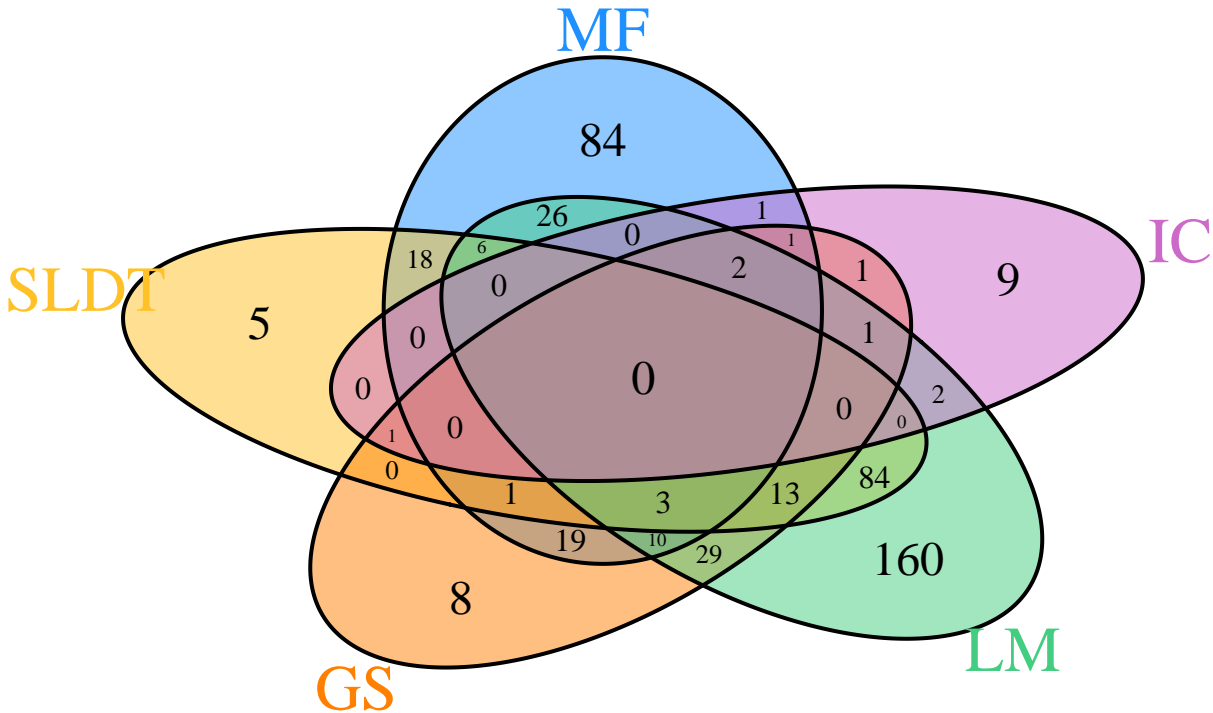

Supplement: Supplementary file 2 — Supplemental Figure 1 [file 41408_2017_44_MOESM2_ESM.pdf]
